# Phenanthrotriazine Derivatives Containing Arylidine Hydrazone Moieties as Novel Potential c-Met Inhibitors with Anticancer Effect

**DOI:** 10.22037/ijpr.2021.114371.14835

**Published:** 2021

**Authors:** Najmeh Edraki, Mohammad Hasan Jamei, Zahra Haghighijoo, Zahra Kayani, Elaheh Raufi, Masoomeh Eskandari, Maryam Firouzi, Hossein Sadeghpour, Ramin Miri, Mehdi Khoshneviszadeh, Omidreza Firuzi

**Affiliations:** a *Medicinal and Natural Products Chemistry Research Center, Shiraz University of Medical Sciences, Shiraz, Iran. *; b *Department of Medicinal Chemistry, School of Pharmacy, Shiraz University of Medical Sciences, Shiraz, Iran.*

**Keywords:** Targeted therapy, Receptor tyrosine kinase inhibitor, Breast cancer, Schiff base, Cell cycle block, Antiproliferative

## Abstract

Cancer is the second cause of death in the world and the discovery of novel anticancer agents is of vital importance to provide better therapeutic options for cancer patients. In this study, a new series of 12 arylidene hydrazone phenanthrotriazine derivatives were designed, synthesized, and tested *in-vitro* for antiproliferative activity against three cancer cell lines including colorectal cancer (HT-29), breast cancer (MCF-7) and leukemia (MOLT-4) cells and also against Vero normal cells. The effect of derivatives on cell cycle and apoptosis induction were studied by flow cytometric propidium iodide/RNase assay and Hoechst 33258 staining, respectively, while docking analysis was used to investigate the interactions of synthesized derivatives with the c-Met receptor kinase domain. Some compounds showed considerable antiproliferative activity against tested cancer cells. The most potent derivative was **9k** bearing pyrrole moiety with IC_50_ values of 14.3, 4.7 and 1.7 µM against HT-29, MCF-7 and MOLT-4 cells, respectively, while it showed negligible activity against Vero normal cells (IC_50_: 95.4 µM). Derivatives bearing 2-nitrophenyl (**9g**), 4-cyanophenyl (**9j**), pyrrole (**9k**), and thiophene (**9l**) moieties induced G0/G1 cell cycle arrest and also apoptosis at higher doses in MCF-7 cells. Docking study showed that the phenanthrotriazine backbone form H-bond interactions with Asn1209, while phenyl moieties of the pendants generate different hydrophobic interactions with the Asp1164 and Asp1231 residues of c-Met. In conclusion, phenanthrene 1,2,4-triazines, especially the ones with less influence on normal cells, may constitute promising compounds for the discovery of antiproliferative agents with potential c-Met inhibitory capacity.

## Introduction

Cancer is one of the most lethal diseases in the world. According to the latest GLOBOCAN report, a project of the International Agency for Research on Cancer (IARC), more than 18 million new cases of cancer and over 9 million cancer-related deaths have been registered in 2018 worldwide (2). There have been considerable improvements in several available pharmacological tools for the management of cancer; however, these therapeutic strategies very often suffer from severe limitations including inadequate efficacy, systemic toxicity, and development of drug resistance(Ya). In this regard, the discovery of novel anticancer agents with improved efficacy and toxicity profiles is a necessity and also a major challenge for the scientific community(T). 

**Figure 1:**
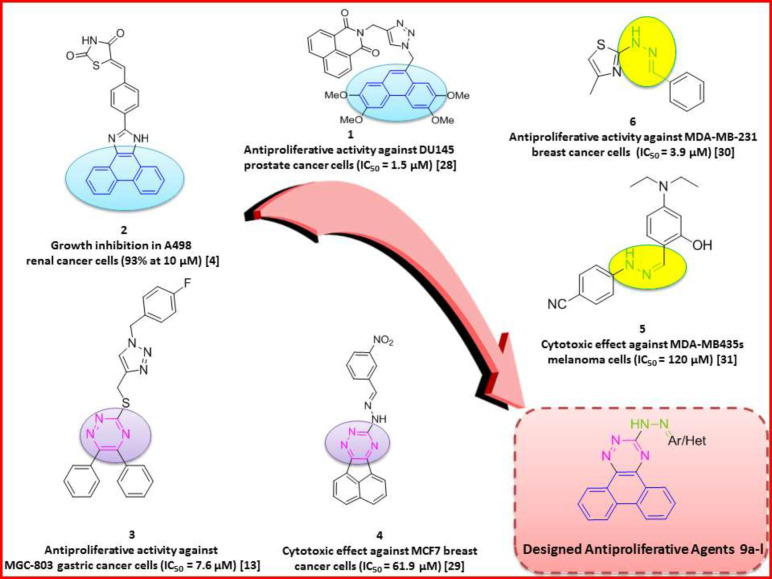
Design strategy of target compounds with arylidine hydrazone phenanthrenotriazine structure as anti-cancer agents

Phenanthrene-based derivatives are well established as antitumor agents (4, 5) showing antiproliferative effects in different cancers including breast cancer (6), non-small cell lung cancer (7, 8), as well as several others (6) (Figure 1). Phenanthroindolizidine and phenanthroquinolizidine derivatives from the alkaloid chemical class of compounds have also been reported as potent anti-tumor agents (9, 10). Phenanthrene derivatives have been reported to exert their anticancer effect by interference with DNA synthesis and intercalation with DNA base pairs (4, 5), interference with the cancer cell cycle (6) and (W,S)activation of apoptotic pathways (6-8, 11).

On the other hand, several hetero-fused 1,2,4-triazine derivatives have shown significant antitumor action against different cancer types (12). These compounds have been reported to induce their effect by several mechanisms including apoptosis induction and cell cycle perturbation in cancer cells (13).

In recent years, molecularly targeted small molecule agents have achieved considerable success in treatment of different types of cancer (14). Receptor tyrosine kinases (RTKs) are frequently found to be altered and overexpressed in several tumor types and they have been linked with certain hallmarks of cancer including proliferation, invasion as well as metastasis (15-17). RTKs are currently considered crucial targets to design small-molecule inhibitors with anticancer potential (18, 19). MET proto-oncogene (c-Met) is one of the well-studied RTKs and several compounds with inhibitory potential against this target are being developed to fight against cancer (20-23). Among several studied chemical scaffolds, the hetero-fused 1,2,4-triazine derivatives have also been reported to inhibit c-Met kinase in previous studies (24-27).

In continuation of our interest in the discovery of novel anticancer agents (28, 29), we focused on structural modifications of previously reported antiproliferative agents including phenanthrene (Figure 1, compounds **1** and **2**) (4, 30) and 1,2,4-triazine (Figure 1, compound **3** and **4)** (13, 31) as innovative backbones to design potent antiproliferative agents. In addition, according to previous reports, hydrazone linkers (Figure 1, compounds **5** and **6**) (32, 33) have also been widely presented as promising cytotoxic structures. Based on these studies, twelve novel derivatives of phenanthrotriazine bearing imine linkers of appropriate aryl pendants were synthesized. The effect of synthesized compounds on human cancer cell proliferation, cell cycle, and induction of apoptosis was investigated. Docking analysis was used to study compounds’ interactions with the c-Met receptor active site.

## Experimental

*Chemistry*

Melting points were determined with a MEL-TEMP model 1202D. FT-IR spectra were recorded with a Bruker Tensor 27 spectrometer as KBR disks. The MS spectra were recorded using Agilent 7000-3Q mass spectrometer at an electron impact mode with an ionization voltage of 70 eV. ^1^H-NMR and ^13^C-NMR spectra were recorded with Bruker Spectrospin Avance 300 and 75 MHz spectrometers. CDCl_3 _and DMSO were used as solvents, while TMS was utilized as an internal standard for chemical characterizations. All chemical shifts were reported as δ (ppm) and coupling constants (J) are given in Hz. Thin-layer chromatography was performed with glass-backed plates (20 × 20 cm^2^, 500 μ) using silica gel (Merk Kieselgel 60 HF_254_, Art. 7739). The chemical reagents used for the synthesis were purchased from Merck and Sigma-Aldrich.

*Synthesis of phenanthro(9,10-e)(1,2,4)triazine 3-thiol (3)*

To a solution of Phenanthrene 9, 10 dione (**1**) (10 mmol) and acetic acid (40 mL), thiosemicarbazide (**2**) (20 mmol) was added and refluxed for 4 h. The reaction progression was monitored using TLC. The precipitated product was filtered and washed with enough amounts of cold water and ethanol mixture. Orange solid; Yield: 95%; mp: 122-124 °C; ^1^H-NMR (300 MHz, CDCl_3_): δ_H_ 14.65 ppm (s, 1H, NH), 8.55 (m, 2H, Ar-H), 8.15 (m, 5H, Ar-H), 7.55 (m, 9H, Ar-H), 6.72 (s, 1H, SH); IR (KBr, cm ^-1^) υ max: 3413 (-NH-), 3145 (ArCH), 1596 (Ar N=N), 1500 (-C=N-), 1481 (Ar C=C); MS (EI) *m/z *(%): 263 (M^+^, 40), 235 (89), 176 (100).

*Synthesis of 3-(methylthio)phenanthro(9, 10-e)(1,2,4)triazine (5)*

Phenanthrotriazine thiol (**3**) (10 mmol) was added to the mixture of NaOH (460 mg, 11.5 mmol) in warm ethanol (50 mL) then the excess amount of methyl iodide (**4**) (15 mmol, 1 mL) was added after the temperature was decreased. The reaction mixture was stirred at room temperature for 3 h. The reaction progress was monitored using TLC. The resulting yellow suspension was filtered through a filtration paper and then washed with enough amounts of cold ethanol. Yellow solid; Yield: 78%; mp: 157-160 °C; ^1^H-NMR (300 MHz, CDCl_3_): δ_H_ 14.65 ppm (s, 1H, NH), 8.55 (m, 2H, Ar H), 8.15 (m, 5H,Ar H), 7.55 (m, 9H, Ar H), 6.72 (s, 1H, SH); IR (KBr, cm ^-1^) υ max:2927 (ArCH), 1605 (Ar N=N), 1503 (-C=N-), 1482 (Ar C=C); MS (EI) *m/z *(%): 263 (M^+^, 40), 235 (89), 176 (100).

*3-(2-benzylidene hydrazinyl)phenanthro(9, 10-e)(1,2,4)triazine(7)*

Methyl thiol (**5**) (2 mmol) was dissolved in 2-propanol and refluxed. Excess amounts of hydrazine hydrate (**6**) (5 mL) were added and the mixture was refluxed overnight and the reaction progression was monitored using TLC. The resulting yellow suspension was filtered through a filtration paper and then washed with enough amounts of cold water. 

Yellow solid; Yield: 77%; IR (KBr, cm ^-1^) υ max: 3444 (-NH-), 3039 (ArCH), 1556 (Ar N=N), 1523 (-C=N-), 1447 (Ar C=C); MS (EI) *m/z *(%): 261 (M^+^, 57), 286 (79), 190 (100).

*General procedure for the synthesis of hydrazones (9a-l)*

Hydrazine (**7**) (0.5 mmol) was dissolved in warm ethanol (20 mL). The appropriate aryl aldehyde (**8**) (0.5 mmol) was added and the reaction mixture was refluxed for 3 h and the reaction progression was monitored using TLC. The resulting yellow suspension was filtered through a filtration paper and then washed with enough amounts of cold ethanol.

*3-(2-benzylidenehydrazinyl)phenanthro [9,10-e][1,2,4]triazine (* ***9a*** *)*

Yellow solid; Yield: 73%; mp: 286-290 °C; ^1^H-NMR (300 MHz, DMSO): δ_H_9.14-9.17 (m, 2H, Ar H),8.83-8.86 (m, 2H, Ar H), 8.40 (s, 1H, =CH), 7.96-7.99 (d, *J *= 9 Hz, 1H, Ar H),7.81-7.89 (m, 5H, Ar H), 7.44-7.54 (m, 3H, Ar H); IR (KBr, cm ^-1^) υ max: 3419 (-NH-), 2983 (ArCH), 1588 (Ar N=N), 1529 (-C=N-), 1447 (Ar C=C); MS (EI) *m/z *(%): 349 (M^+^, 24), 272 (28), 218 (100), 190 (96).

*3-(2-(2-methoxybenzylidene)hydrazinyl)phenanthro[9,10-e][1,2,4]triazine (* ***9b*** *)*

Yellow solid; Yield: 76%; mp: 252-254 °C; ^1^H-NMR (300 MHz, DMSO): δ_H_9.12-9.16 (m, 2H, Ar H),8.77-8.82 (m, 2H, Ar H), 8.75 (s, 1H, =CH),8.04-8.07 (d, *J* = 9Hz, 1H, Ar H),7.93-7.97 (m, 1H, Ar H), 7.78-7.83 (m, 3H, Ar H), 7.40-7.45 (t, *J* = 9Hz, 1H, ArH), 7.07-7.15 (m, 2H, Ar H), 3.90 (s, 3H, CH_3_); IR (KBr, cm ^-1^) υ max: 3445 (-NH-), 2977 (ArCH), 1584 (Ar N=N), 1522 (-C=N-), 1447 (Ar C=C); MS (EI) *m/z *(%): 379 (M^+^,29), 272 (14), 246 (37), 218 (100), 190 (76).

*3-(2-(4-methoxybenzylidene)hydrazinyl)phenanthro[9,10-e][1,2,4]triazine (* ***9c*** *)*

Yellow solid; Yield: 76%; mp: 288-292 °C; ^1^H-NMR (300 MHz, DMSO): δ_H_9.14-9.16 (m, 2H, Ar H),8.83-8.85 (m, 2H, Ar H), 8.34 (s, 1H, =CH),7.94-7.99 (m, 1H, Ar H),7.76-7.83 (m, 5H, Ar H), 7.06-7.08 (d, *J* = 6 Hz, 2H, Ar H), 3.83 (s, 3H, CH_3_); IR (KBr, cm ^-1^) υ max: 3445 (-NH-), 2989 (ArCH), 1611 (Ar N=N), 1526 (-C=N-), 1430(Ar C=C); MS (EI) *m/z *(%): 379 (M^+^, 12), 245 (27), 218 (100), 190 (55).

*2-((2-(phenanthro[9,10-e][1,2,4]triazin-3-yl)hydrazono)methyl)phenol (* ***9d*** *)*

Yellow solid; Yield: 61%; mp: 328-332 °C; ^1^H-NMR (300 MHz, DMSO): δ_H_9.09-9.15 (m, 2H, Ar H),8.84-8.87 (m, 2H, Ar H), 8.56 (s, 1H, =CH),7.97-8.02 (t, *J *= 9 Hz, 1H, Ar H),7.81-7.91 (m, 3H, Ar H), 7.51-7.57 (d, *J* = 6 Hz, 1H, Ar H), 7.30-7.36 (t, *J* = 7.5 Hz, 1H, Ar H), 6.97-7.05 (m, 2H, Ar H); IR (KBr, cm ^-1^) υ max: 2966 (ArCH), 1585 (Ar N=N), 1525 (-C=N-), 1430 (Ar C=C); MS (EI) *m/z *(%): 365 (M^+^, 45), 218 (100), 190 (92).

*3-((2-(phenanthro[9,10-e][1,2,4]triazin-3-yl)hydrazono)methyl)phenol (* ***9e*** *)*

Yellow solid; Yield: 64%; mp: 219-223 °C; ^1^H-NMR (300 MHz, DMSO): δ_H_9.06-9.14 (m, 3H, Ar H),8.78 (s, 1H, =CH), 8.71-8.76 (m, 2H, Ar H),7.90-7.94 (t, *J *= 7.2 Hz, 2H, Ar H),7.72-7.80 (m, 5H, Ar H), 4.63 (s, 1H, NH); IR (KBr, cm ^-1^) υ max: 3445 (-NH-), 3047 (ArCH), 1556 (Ar N=N), ~1530 (-C=N-), 1447 (Ar C=C); MS (EI) *m/z *(%): 365 (M^+^, 16), 286 (27), 272 (31), 218 (85), 190 (100).

*3-((2-(phenanthro[9,10-e][1,2,4]triazin-3-yl)hydrazono)methyl)benzene-1,2-diol (* ***9f*** *)*

Yellow solid; Yield: 57%; mp: 329-333 °C; ^1^H-NMR (300 MHz, DMSO): δ_H_9.11-9.19 (m, 2H, Ar H),8.80-8.87 (m, 2H, Ar H), 8.52 (s, 1H, =CH),7.98-8.03 (t, *J *= 7.2 Hz, 1H, Ar H),7.81-7.90 (m, 3H, Ar H), 6.97-7.00 (d, *J* = 6 Hz, 1H, Ar H), 6.88-6.90 (d, *J* = 6 Hz, 1H, Ar H), 6.76-6.81 (t, *J* = 9 Hz, 1H, Ar H); IR (KBr, cm ^-1^) υ max: 3495 (-NH-), 2851 (ArCH), 1596 (Ar N=N), 1525 (-C=N-), 1470 (Ar C=C); MS (EI) *m/z *(%): 381 (M^+^, 80), 364 (21), 218 (99), 190 (100).

*3-(2-(2-nitrobenzylidene)hydrazinyl)phenanthro[9,10-e][1,2,4]triazine (* ***9g*** *)*

Yellow solid; Yield: 70%; mp: 225-229 °C; ^1^H-NMR (300 MHz, DMSO): δ_H_8.98-9.15 (m, 3H, Ar H),8.75 (s, 1H, =CH), 8.68-8.72 (m, 2H, Ar H),7.87-7.97 (m, 2H, Ar H),7.72-7.84 (m, 5H, Ar H), 4.61 (s, 1H, NH); IR (KBr, cm ^-1^) υ max: 3445 (-NH-), 3038 (ArCH), 1643 (-NO_2_), 1556 (Ar N=N), 1524 (-C=N-), 1447 (Ar C=C); MS (EI) *m/z *(%): 395 (M^+^, <10), 261 (100), 233 (18), 190 (82).

*3-(2-(3-nitrobenzylidene)hydrazinyl)phenanthro[9,10-e][1,2,4]triazine (* ***9h*** *)*

Yellow solid; Yield: 57%; mp: 216-219 °C; ^1^H-NMR (300 MHz, DMSO): δ_H_9.05-9.19 (m, 3H, Ar H),8.77 (s, 1H, =CH), 8.70-8.74 (m, 2H, Ar H),7.86-7.97 (t, *J *= 6.9 Hz, 2H, Ar H),7.74-7.83 (m, 5H, Ar H), 4.62 (s, 1H, NH); IR (KBr, cm ^-1^) υ max: 3444 (-NH-), 3038 (ArCH), 1644 (NO_2_), 1556 (Ar N=N), 1523 (-C=N-), 1447 (Ar C=C); MS (EI) *m/z *(%): 261 (94), 190 (100), 175 (44), 205(21), 233(17).

*3-(2-(4-nitrobenzylidene)hydrazinyl)phenanthro[9,10-e][1,2,4]triazine (* ***9i*** *)*

Yellow solid; Yield: 58%; mp: 240-244 °C; ^1^H-NMR (300 MHz, DMSO): δ_H_9.05-9.12 (m, 3H, Ar H),8.77 (s, 1H, =CH), 8.71-8.74 (m, 2H, Ar H), 7.89-7.94 (t, *J *= 7.2 Hz, 2H, Ar H),7.74-7.79 (m, 5H, Ar H), 4.63 (s,1H, NH); IR (KBr, cm ^-1^) υ max: 3444 (-NH-), 3046 (ArCH), 1556 (Ar N=N), 1523 (-C=N-), 1447 (Ar C=C); MS (EI) *m/z *(%): 394 (M^+^, 25), 272 (34), 218 (79), 190 (100).

*4-((2-(phenanthro[9,10-e][1,2,4]triazin-3-yl)hydrazono)methyl)benzonitrile (* ***9j*** *)*

Yellow solid; Yield: 54%; mp: 231-234 °C; ^1^H-NMR (300 MHz, DMSO): δ_H_9.03-9.11 (m, 3H, Ar H),8.76 (s, 1H, =CH), 8.71-8.75 (m, 2H, Ar H),7.87-7.93 (t, *J *= 9Hz, 2H, Ar H),7.72-7.78 (m, 5H, Ar H), 4.63 (s, 1H, NH); IR (KBr, cm ^-1^) υ max: 3444 (-NH-), 3035 (ArCH), 2226 (CN), 1556 (Ar N=N), 1523 (-C=N-), 1447 (Ar C=C); MS (EI) *m/z *(%): 374 (M^+^, 28), 272 (35), 218 (81), 190 (100).

*3-(2-((1H-pyrrol-2-yl)methylene)hydr -zinyl)phenanthro[9,10-e][1,2,4]triazine (* ***9k*** *)*

Yellow solid; Yield: 63%; mp: 210-214 °C; ^1^H-NMR (300 MHz, DMSO): δ_H_9.02-9.12 (m, 3H, Ar H),8.76 (s, 1H, =CH), 8.71-8.73 (m, 2H, Ar H),7.88-7.96 (t, *J *= 6.9 Hz, 1H, Ar H),7.74-7.85 (m, 5H, Ar H); IR (KBr, cm ^-1^) υ max:3439 (-NH-), 2917 (ArCH), 1556 (Ar N=N), 1524 (-C=N-), 1447 (ArC=C); MS (EI) *m/z *(%): 338 (M^+^, <10), 261 (100), 190 (84), 176 (38).

3-(2-(thiophene-2-ylmethylene)hydraz-inyl)phenanthro[9,10-e][1,2,4]triazine (**9l**)

Yellow solid; Yield: 40%; mp: 228-231 °C; ^1^H-NMR (300 MHz, DMSO): δ_H_9.07-9.11 (m, 3H, Ar H),8.75 (s, 1H, =CH), 8.66-8.73 (m, 2H, Ar H),7.87-7.92 (t, *J *= 8.1 Hz, 1H, Ar H),7.67-7.83 (m, 5H, Ar H), 4.61 (s, 1H, NH); IR (KBr, cm ^-1^) υ max: 3445 (-NH-), 1556 (Ar N=N), 1524 (-C=N-), 1447 (Ar C=C); MS (EI) *m/z *(%): 355 (M^+^, 14), 246 (31), 218 (100), 190 (81).

*Evaluation of the antiproliferative effect*

Fetal bovine serum (FBS), phosphate-buffered saline (PBS), RPMI-1640, and trypsin were obtained from Biosera (Ringmer, UK). Penicillin/streptomycin and MTT (3-(4,5-dimethylthiazol-2-yl)-2,5-diphenyltetrazolium bromide) were from Sigma-Aldrich (Saint Louis, MO, USA) and Invitrogen (San Diego, CA, USA), respectively. Dimethyl sulfoxide and doxorubicin were purchased from Merck (Darmstadt, Germany) and EBEWE Pharma (Unterach, Austria), respectively. The cells were acquired from Iranian Biological Resource Center, Tehran, Iran (HT-29 (human colorectal adenocarcinoma), MCF-7 (human breast adenocarcinoma) and Vero (African green monkey kidney) cells) or National Cell Bank of Iran, Pasteur Institute, Tehran, Iran (MOLT-4 (lymphoblastic leukemia) cells). 

MTT reduction assay was employed to assess the viability of cancer cells after being treated with synthesized derivatives as previously described (34). The cells were plated in flat-bottom 96-well microplates, at densities of 3 × 10^4^ cells/mL (100 μL in each well) and then incubated overnight at 37 °C. Three to four different concentrations of synthesized derivatives (in the range of 1 to 100 μM) were placed in triplicate wells and the plates were incubated for 72 h at 37 °C. The concentration of DMSO did not exceed 0.25% in each well. At the end of the incubation time, 80 μL of the media in each well was replaced with the same amount of growth medium without phenol red containing 0.5 mg/mL of MTT. Cells were further incubated at 37 °C for 4 h and then 80 μL of the solution of each well was removed. The addition of DMSO (200μL) to each well solubilized the formazan crystals formed inside the viable cells after 1-hour incubation followed by 30-minute shaking. The optical absorbance of the final solution was determined at 570 nm using a Bio-Tek multimode plate reader (Synergy HTX). The cell viability was calculated compared to untreated control cells by comparison of absorbance measurements. CurveExpert software (version 1.34 for Windows) was used to determine the IC_50_ value for each compound. To confirm the results, every experiment was repeated 3 to 5 times. 

*Cell Cycle Analysis*

Alterations of the cell cycle were determined by the propidium iodide (PI)-RNase flow cytometric method (35). MCF-7 cells were used to determine the percentage of cells in each phase of the cell cycle. In 6-well plates, 2 × 10^5^ cells were seeded in each well and incubated at 37 °C for 48 h. Afterward, they were treated for 48 h with derivatives **9g**,** 9j**,** 9k **and **9l** at 10 and 25 μM. The cells were then collected after trypsinization, washed twice with PBS, and fixed by 5 mL ethanol 70% for at least 24 h at -20 °C. After fixation, MCF-7 cells were washed with PBS two times and stained with PI 20 µg/mL and RNase 200 µg/mL for 30 min in the dark at room temperature. A FACS Calibur ﬂow cytometer (BD Biosciences) was used for the assays and 20,000 events were analyzed. The number of cells distributed in sub-G1, G0/G1, S, and G2/M were determined by CellQuest software (BD, USA). 

*Hoechst 33258 staining*

Hoechst 33258 was used as a DNA stain to detect apoptosis in cells. MCF-7 cells were seeded in a 6-well plate at a density of 5 × 10^4^ cells/mL (2 mL per well) and incubated for 24 h. The whole medium was removed and different concentrations of test compounds **9g**,** 9j**,** 9k, **and **9l** diluted in 2 mL medium were placed in the different wells and incubated for 72 h. Afterward, the medium was removed again, 1 mL of 4% cold freshly prepared paraformaldehyde (PFA) was added, and cells were incubated for 20 min at room temperature. The cells were then washed 2 times with 1 ml PBS and incubated with 1 mL Hoechst 33258 2.5 µg/mL for 30 min in the dark at room temperature (36, 37). In the end, the cells were washed with 1 mL PBS and imaged with a fluorescence microscope.

*Docking analysis*

The two most potent compounds (**9k** and **9l**) were prepared using MarvinSketch 18.20.0 and geometry optimization was carried out by the steepest descent algorithm with MOPAC. The crystal structure of c-Met kinase receptor in complex with selective a c-Met inhibitor was obtained from the RCSB Protein Data Bank (PDB code 3ZZE). The molecular docking study was done with a GOLD software package using the GoldScore and ChemPLP scoring functions and default settings. The active site of protein was defined for the protein residues within 8 Å of the cognate ligand that accompanied the downloaded protein complexes. The cognate ligand was docked inside the 3ZZE to validate the docking algorithm using both scoring functions. Re-docking of the cognate ligand resulted in RMSD values of 0.854 Å and 1.01Å, for GoldScore and ChemPLP, respectively. Thus, the score value of the interactions between the ligands and c-Met kinase receptors with PDB code 3ZZE was calculated in terms of the GoldScore fitness function. The GoldScore fitness value for cognate ligand was calculated as 32.50. The molecular models of the docked compounds were visualized in the BIOVIA Discovery Studio software package and Pymol Software 1.7.4.4 Edu.

## Results and Discussion

*Chemistry*

**Scheme 1:**
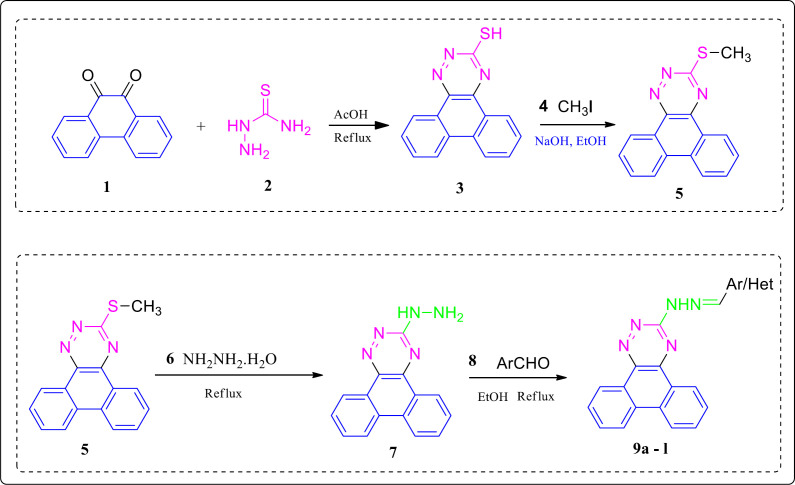
Synthesis procedure of phenanthrotriazine derivatives

The synthesis route of the compounds **9a-l** is depicted in Scheme 1. Firstly, the starting material phenanthrene 9, 10 dione **1** was treated with thiosemicarbazide **2** in acetic acid (40 mL) conditions to give phenanthro(9,10-e)(1,2,4)triazine 3-thiol **3** with a relatively high yield (95%). Subsequently, methylation of the thiol **3** through a nucleophilic substitution reaction was accomplished using methyl iodide **4** to afford 3- methylthio)phenanthrotriazine derivative **5** (yield 78%). The hydrazinolysis of the methylthiol intermediate **5** was done via a nucleophilic substitution reaction in the presence of hydrazine hydrate to form the hydrazine product **7**. In the final step, several aryl aldehydes were attached to the hydrazine phenanthrotriazine **7** intermediate via an imine linkage to give the corresponding phenanthrotriazine hydrazones products **9a-l** in 40–76% yields. The ^1^H NMR spectra of all compounds were in good agreement with the structures, appearing at comparable chemical shifts with those reported for other hydrazones with different substituted (31). 

*Evaluation of the anticancer activity*

The antiproliferative effect of newly synthesized compounds was assessed against three human cancer cell lines including HT-29 (colorectal adenocarcinoma), MCF-7 (breast adenocarcinoma) and MOLT-4 (lymphoblastic leukemia) cells as well as Vero (African green monkey kidney) cells, a normal cell line frequently used as non-cancer reference cells by several investigators (38-40). Doxorubicin and cisplatin were also tested as standard cytotoxic agents (Table 1).

Compounds **9a** and **9f** did not lower cell viability below 50% at the highest tested concentrations (25 µM). Two analogs including **9c **and **9d** were not tested due to the very low solubility in aqueous media. The remaining 8 compounds among 12 tested derivatives, displayed considerable antiproliferative effects against the cancer cell lines with IC_50_ values ranging 7.6-20.1, 4.7-17.7 and 1.7-7.8 µM, against HT-29, MCF-7 and MOLT-4 cells, respectively. 

The highest antiproliferative effects against MCF-7 and MOLT-4 cells were observed by compound **9k** containing pyrrole moiety with IC_50 _values of 4.5 ± 0.16 and 1.7 ± 0.2 µM, respectively, while the highest potency against HT-29 cells was demonstrated by **9i **bearing *para* nitrophenyl group with an IC_50_ value of 7.6 ± 0.8 µM. Derivative **9k**, showed negligible effect against normal Vero cells with an IC_50_ value of 95.4 ± 12.4 µM, while derivative **9i** was almost equally effective against non-cancer cells with an IC_50_ value of 7.4 ± 1.4 µM. 

In a previous report, the antiproliferative effect of a series of phenanthro[9,10-d]imidazole/oxazole and acenaphtho[1,2-d]imidazole derivatives were evaluated against the NCI-60 panel of cancer cell lines and it was similarly shown that these derivative possess antiproliferative activity against different cancer cell lines including breast and colon cancer as well as leukemia, among others (4).

Our findings showed that overall the compounds had similar activities against 3 tested cell lines. Considering the influence of different substitutions on the antiproliferative activity, the following structure-activity relationships can be presented:

The presence of heterocyclic rings may enhance the anticancer effect of the derivative against all three cell lines, as shown for derivatives **9k** and **9l** containing pyrrole and thiophene moieties, respectively. The effect of pyrrole substitution in compound **9k** was particularly interesting, because it conferred a high anticancer effect, while it caused an almost entire sparing of normal cells.

The presence of CN (as in **9j**) and NO_2 _(as in **9g**, **9h** and **9i**) groups on the phenyl ring lead to the enhancement of the antiproliferative activity against cancer cells probably by the establishment of H-bonding and polar interactions. Among these derivatives, **9h** was less effective against non-cancer cells. Derivatives **9g** and **9j** had higher IC_50_ values against normal cells compared to breast cancer (MCF-7) and leukemia (MOLT-4) cells, but not compared to colon cancer (HT-29) cells.

Introduction of OCH_3_ moiety (as in **9b**) seemed to lower antiproliferative effect against cancer cells, increasing the IC_50_ value especially against MCF-7 (15.2 ± 3.1 µM).

Compounds **9g, 9j, 9k **and **9l** possessing high activities, each one containing different moieties including nitrophenyl, cyanophenyl, pyrrole and thiophene, respectively, were selected for further analysis of cell cycle alterations, apoptosis, and interaction with the c-Met receptor.

*Cell cycle analysis*

**Figure 2:**
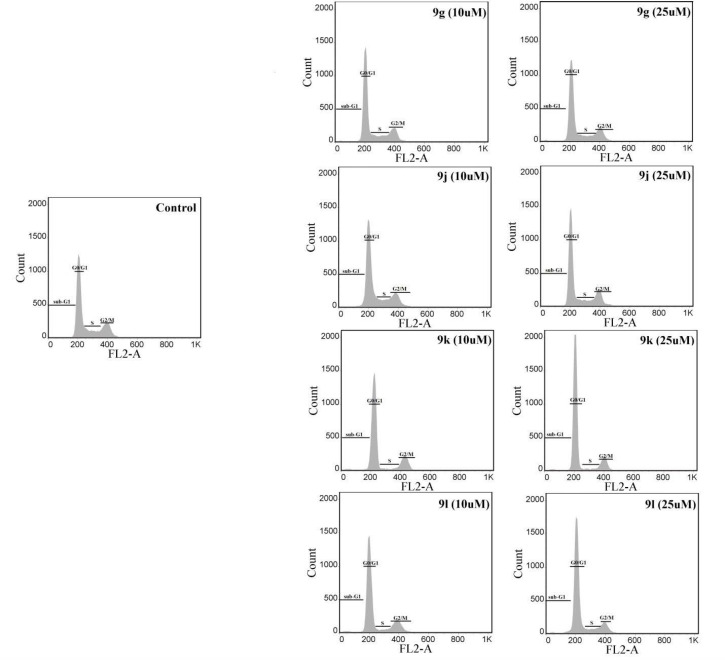
Representative cell cycle histograms of MCF-7 cells treated with synthesized derivatives. Analysis of the percentage of cells in different phases of cell cycle was performed using propidium iodide (PI)-RNase flow cytometric assay. MCF-7 cells were seeded in 6-well microplates and treated with synthesized derivatives for 48 h. The cells were then collected and fixed in 70% ethanol overnight at -20 °C. In the end, the cells were stained with DNA staining solution (PI 20 µg/mL and RNase 200 µg/mL) for 30 min and 20,000 events were analyzed by a FACS Calibur ﬂow cytometer (BD Biosciences). Representative histograms of cells treated with compounds **9g**, **9j**, **9k** and **9l** are shown

The influence of compounds **9g, 9j, 9k **and **9l** on the MCF-7 cancer cell cycle progression was investigated to gain more information on the mechanism of action of derivatives. The cells were treated with 10 and 25 µM concentrations of each derivative for 48 h. Percentages of cells in sub-G1, G0/G1, S and G2/M phases of the cell cycle after being treated with the test compounds are shown in Table 2 and representative histograms are displayed in Figure 2. All four compounds significantly enhanced the number of cells in the G0/G1, while they decreased the percentage of cells in S and G2/M at both concentrations of 10 and 25 µM. Derivatives** 9g, 9k **and **9l **at 25 µM also increased the cells in the sub-G1 phase, which is an index of apoptosis induction in cancer cells. 

A recent study has shown the antiproliferative effect of a series of fifteen 1,2,3-triazolo-phenanthrene derivatives against various cancer cell lines including prostate, breast, lung, gastric, cervical cancer cells. The effect of one of the most potent compounds with an IC_50 _value of 1.5 ± 0.09 µM against DU-145 prostate cancer cells was examined on the alterations of cell cycle distribution in these cells and it was found that indeed this derivative considerably increased the percentage of cells in G0/G1 phase, while decreasing the cells in S and G2/M phase (30). Another previous report has similarly shown that aminopyridazinone derivatives could also arrest SH-SY5Y neuroblastoma cells in G0/G1 phase (41). 

*Hoechst 33258 staining*

**Figure 3:**
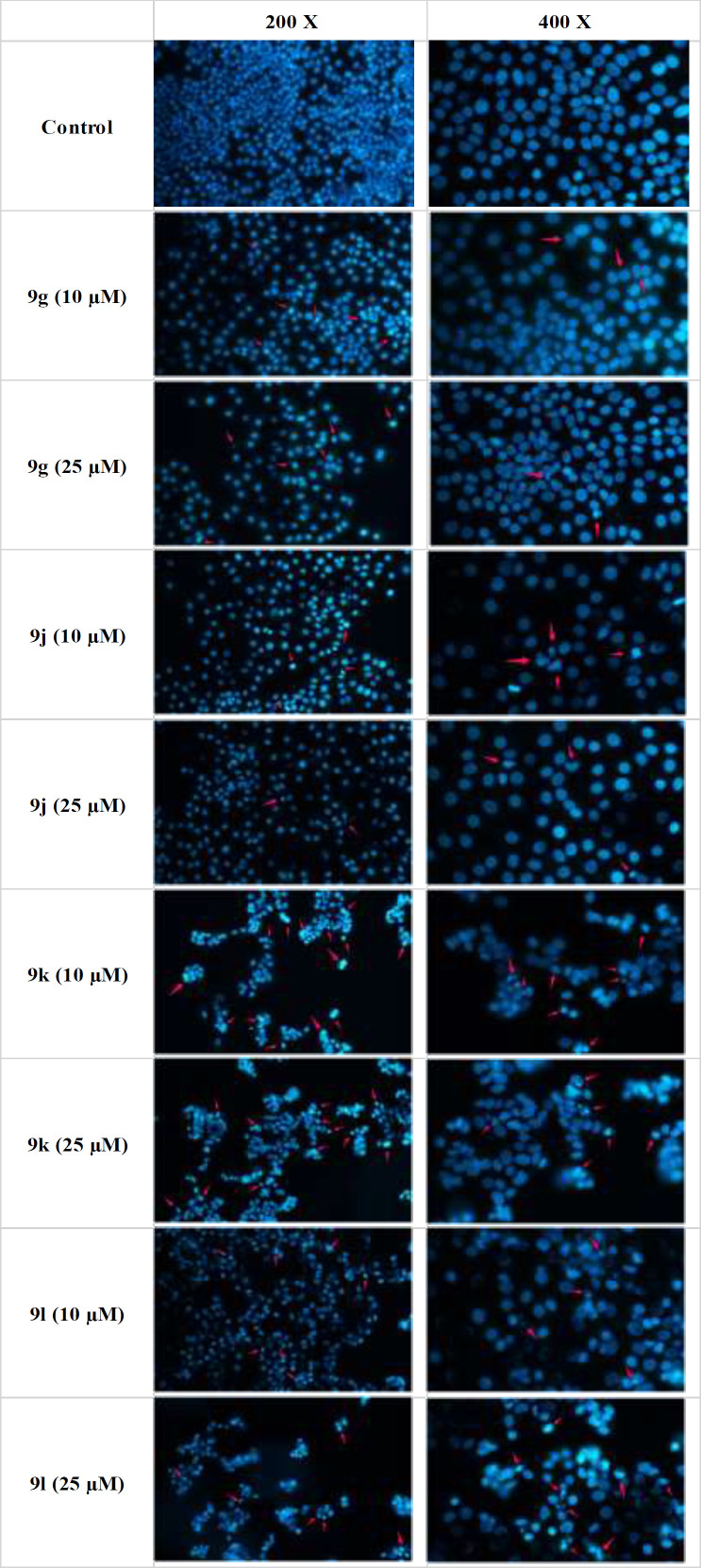
Detection of apoptosis in MCF-7 cells by Hoechst staining. Hoechst 33258 was used as a DNA stain to detect apoptosis in MCF-7 cells treated with synthesized compounds. The cells were seeded in a 6-well plates and treated with different concentrations of test compounds 9g, 9j, 9k and 9l for 72 h. After removing the whole medium, 4% cold freshly prepared paraformaldehyde (PFA) was added for 20 min at room temperature, the cells were then washed twice with PBS, and incubated with Hoechst 33258 2.5 µg/mL for 30 min at room temperature in the dark. Finally, the cells were washed with PBS again and the plates were imaged with a fluorescence microscope

Apoptosis induction in MCF-7 cells upon 72-hour treatment with compounds **9g, 9j, 9k **and **9l** was measured by Hoechst 33258 staining (Figure 3). Hoechst 33258 is a stain that by specifically binding to DNA, can characterize the morphological changes of apoptotic cells, including nuclear shrinkage and fragmentation. Cells treated with all 4 select derivatives, especially **9k** and **9l** at 25 µM showed signs of apoptosis. Consistent with the moderate increase in the sub-G1 phase of the cell cycle, also here we observed that only at higher concentrations and after being exposed for a longer time (72 h) mild to moderate levels of apoptosis could be observed.

*Computational studies and binding mode analysis*

Docking studies of all the synthesized derivatives were performed by GOLD software to investigate their binding modes. It has been shown that hetero-fused 1,2,4-triazine derivatives inhibit Met kinase and exert antiproliferative effects in breast, lung and liver cancer cells (24, 25). Previous studies have also reported that c-Met kinase inhibitors may block the cell cycle progression in G0/G1 phase and also induce apoptosis at higher doses and extended periods of incubation time (42, 43). 

**Figure 4:**
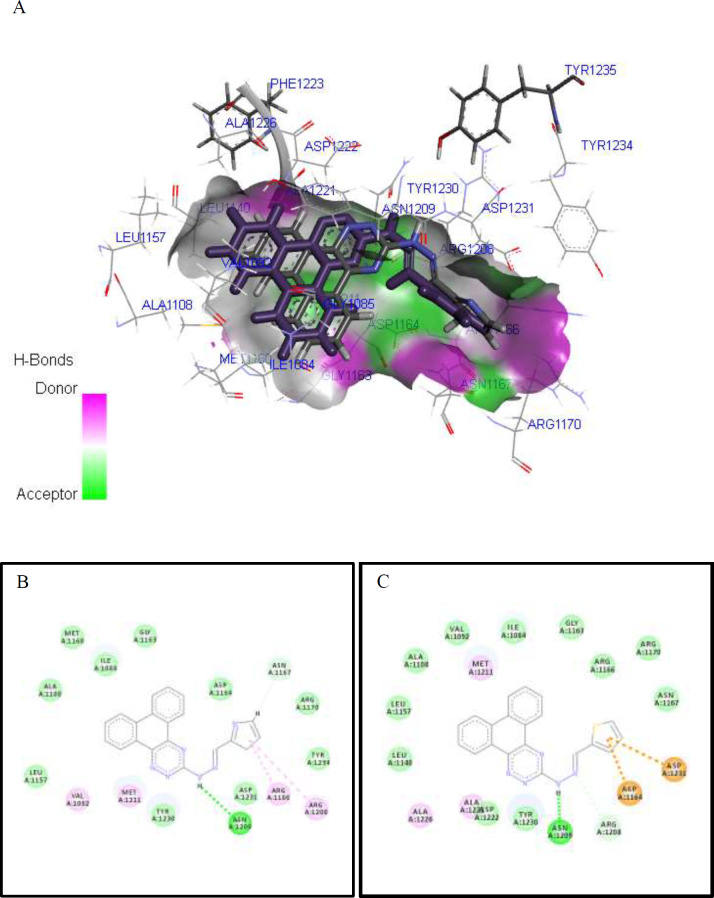
Molecular docking study of the interactions of synthesized derivatives with c-Met receptor active site. Interactions and binding modes of compounds **9k** and **9l** with the c-Met receptor active site (PDB code: 3ZZE) were studied by molecular docking. 3D Stereo view of the overlaid model (A), 2D docking models and possible interactions of **9k** (B) **9l** (C) are shown

The obtained GOLD Score value and the key interactions of all compounds with the c-Met receptor (PDB code: 3ZZE) are presented in Table 3. It is generally expected that a c-Met inhibitor must form several hydrogen bonds and hydrophobic interactions with the kinase domain of the receptor based on the type of inhibitors: type I inhibitors occupy the ATP site, while type II inhibitors occupy the adjacent allosteric site in addition to the ATP-binding pocket (44, 45). For the selected most active compounds **9k** and **9l**, complete models of the interaction in 3D and 2D views of c-Met kinase domain are displayed in Figures 4A-4C. Accordingly, we found an H-bond interaction between the backbone of phenanthrotriazine and Asn1209. Furthermore, different interactions including VDW and Pi-interactions between heteroaromatic moiety of pendants and the 4 residues Arg1208, Arg1166, Asp1164, and Asp1231 were clearly observed. Comparing the best poses found for derivatives **9k** and **9l**, it was revealed that they might mimic type I kinase inhibitors, since the amino acid residues around the bound type I ligands are generally Asp1164, Ile1084, Gly1085 (44).

It has been previously reported that 1,2,4-triazine derivatives may have an inhibitory potential against c-Met. Based on the results obtained by El-Wakil and colleagues, an optimization on 1,2,4-triazine scaffold and conjugation with mercaptopurine led to the identification of a potent c-Met kinase inhibitor derivative, which showed higher activity compared to reference compounds foretinib and BMS-777607 against A549, HT-29 and MKN-45 cancer cells lines. The binding mode analysis revealed similar key interactions by hydrogen bonds (Met 1160, Lys 1110, and Asp 1222) with other lead compounds from these series (27). 

**Table 1:** Antiproliferative activities of synthesized derivatives against cancer cells assessed by MTT reduction assay

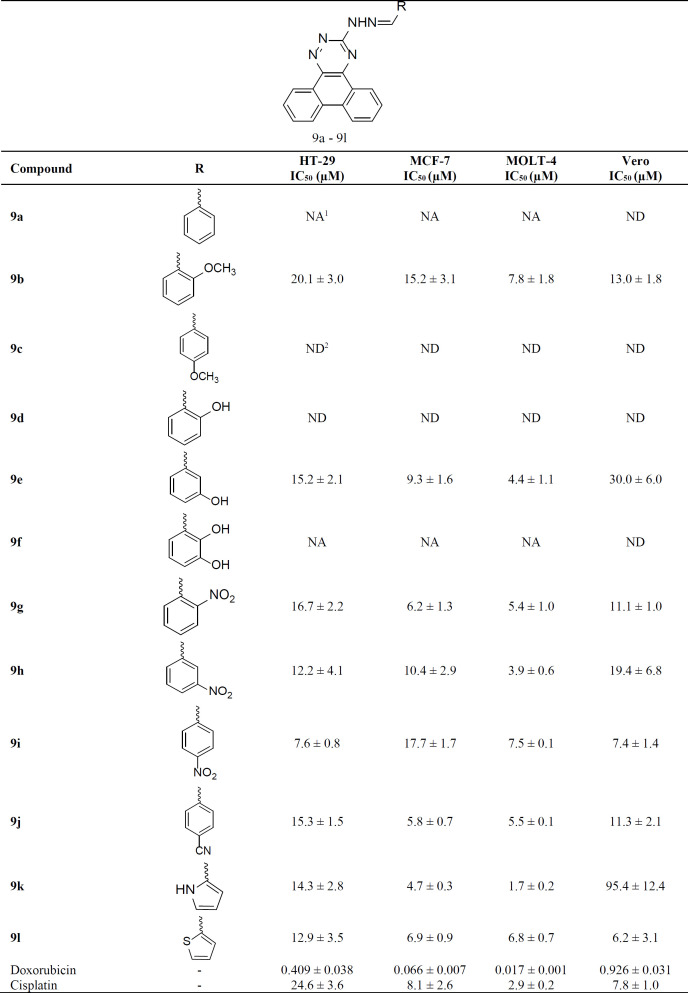

**Table 2:** Effect of synthesized derivatives on distribution of MCF-7 cells in different phases of cell cycle assessed by propidium iodide/RNase flow cytometric assay

**Sample**	**Sub G1**	**G0/G1**	**S**	**G2/M**
Control	0.93 ± 0.1	61.33 ± 1.0	14.35 ± 0.9	23.14 ± 1.0
**9g (10 µM)**	1.35 ± 0.3	69.25 ± 2.1^*^	8.79 ± 1.3^*^	18.58 ± 2.2
**9g (25 µM)**	1.82 ± 0.3^*^	74.76 ± 4.9^*^	7.58 ± 1.9^*^	13.97 ± 3.3^*^
**9j (10 µM)**	1.08 ± 0.2	71.40 ± 2.2^*^	9.59 ± 2.0^*^	17.95 ± 3.0
**9j (25 µM)**	1.30 ± 0.4	73.10 ± 3.0^*^	8.09 ± 2.0^*^	17.51 ± 2.6^*^
**9k (10 µM)**	1.31 ± 0.1	77.30 ± 2.8^*^	4.63 ± 0.7^*^	16.63 ± 2.5^*^
**9k (25 µM)**	1.69 ± 0.4^*^	77.42 ± 3.7^*^	4.68 ± 1.2^*^	16.13 ± 2.9^*^
**9l (10 µM)**	1.32 ± 0.3	73.76 ± 0.6^*^	6.28 ± 1.0^*^	18.50 ± 1.7^*^
**9l (25 µM)**	1.56 ± 0.3^*^	72.07 ± 0.9^*^	6.75 ± 0.9^*^	19.43 ± 0.8^*^

^*^The difference with control was significantly different (*P* ˂ 0.05).

**Table 3:** Results of the computational study of synthesized derivatives docked into the c-Met protein active site (PDB code 3ZZE).

**Compound**	**Gold Score**	**Interaction of the backbone with c-Met**	**Interaction of the pendant with c-Met**
**9a**	59.66	H bond: Asn1209, Arg1208	Pi-anion:Asp1164, Asp1231
**9b**	59.59	H bond: Asn1209, Arg1208	Pi-anion:Asp1164, Asp1231
**9c**	62.71	H bond: Asn1209	H bond: Asp1164, Arg1166 Pi-anion: Asp1231
**9d**	54.33	H bond: Asn1209	H bond: Asp1164 Pi-anion: Asp1231
**9e**	57.14	H bond: Met1160, pro1158	-
**9f**	58.51	H bond: Asn1209	H bond: Arg1170, Arg1166 Pi-anion:Asp1164, Asp1231
**9g**	51.15	H bond: Asn1209	Pi-anion:Asp1164, Asp1231
**9h**	50.29	-	Pi-pi T shaped: Tyr1230
**9i**	55.89	Pi staked:Tyr1230	H bond: Met1160
**9j**	57.73	H bond: Asn1209 Pi staked:Tyr1230	Hydrophobic: Arg1208, Arg1166
**9k**	61.53	H bond: Asn1209 Hydrophobic: Arg1208	Pi-anion: Asp1164, Asp1231
**9l**	50.37	Pi-pi T shaped: Tyr1230	Hydrophobic: Arg1208
**Crizotinib**	60.59	Met1160, Asp1222, Tyr1230, Arg1208	

## Conclusion

We designed a 4-step reaction protocol to synthesize novel chemicals with phenanthrene triazole scaffold under conveniently available conditions. Most synthesized compounds showed *in vitro* antiproliferative effects against colorectal cancer, breast cancer, and leukemia cells as determined by the MTT assay. Four compounds including **9g, 9j, 9k **and **9l** possessing high antiproliferative activities, containing 2-nitrophenyl, 4- cyanophenyl, pyrrole and thiophene moieties, respectively, were selected for further studies. Compound **9K** showed an almost negligible effect on non-cancer cells. Derivatives **9g** and **9j** had higher IC_50_ values against normal cells compared to MCF-7 and MOLT-4 cells, but not compared to HT-29 cells. Analysis of cell cycle alterations revealed that the derivatives significantly increased the percentage of cells in the G0/G1 phase of the cell cycle and also the sub-G1 phase at higher concentrations in MCF-7 cells. Apoptosis assay revealed that derivatives induced moderate levels of apoptosis in the same cells. These effects on the cell cycle and induction of apoptosis at higher doses and longer incubations times was consistent with other reports on the biological effects of RTK inhibitors including c-Met blocking agents. Based on these observations and also the structural similarity of the synthesized derivatives with previously reported c-Met inhibitors, we hypothesized that these compounds may interact with this RTK and examined the interaction of active compounds with the active site of c-Met. The findings of the docking study showed that most of the compounds, especially the ones with higher potencies, showed reasonable interactions with the c-Met kinase domain.

Overall, the findings of this study show that compounds with phenanthrotriazine structure possess considerable anticancer effects and this activity may be due to their inhibitory potential against the c-Met receptor. Hence, these compounds, especially the ones with low activity against normal cells, represent promising agents as molecularly targeted small molecules to fight against cancer.
